# Proteomics investigation of the changes in serum proteins after high- and low-flux hemodialysis

**DOI:** 10.1080/0886022X.2018.1491406

**Published:** 2018-10-04

**Authors:** Shuai Han, Kaiguang Yang, Hong Zhu, Jianhui Liu, Lihua Zhang, Jiuyang Zhao

**Affiliations:** a Affiliated Zhongshan Hospital of Dalian University, Dalian, China;; b Key Laboratory of Separation Science for Analytical Chemistry, Dalian Institute of Chemical Physics, Chinese Academy of Science, National Chromatographic Research and Analysis Center, Dalian, China;; c Department of Nephritic Medicine, Second Affiliated Hospital of Dalian Medical University, Dalian, China

**Keywords:** Proteomics, serum proteins, hemodialysis, dialyzer

## Abstract

**Purpose:** This study aimed to use proteomics methods to investigate the changes in serum protein levels after high- and low-flux hemodialysis (HD).

**Methods:** Before and after HD, serum samples were obtained from two selected patients who were treated with a Polyflux 140 H high-flux dialyzer and a Polyflux 14 L low-flux dialyzer during two continuous therapy sessions. Liquid chromatography-tandem mass spectrometry (LC-MS/MS) was performed to identify the proteins.

**Results:** A total of 212 and 203 serum proteins were identified after high-flux and low-flux HD, respectively. After high-flux HD, 21 proteins increased, and 132 proteins decreased. After low-flux HD, 87 proteins increased, and 45 proteins decreased. High-flux HD led to a significantly greater reduction in protein levels than low-flux HD (0.73 ± 0.13 vs. 0.84 ± 0.18, *p* = .00). Among the increased and decreased proteins, the isoelectric point (pI) values mainly ranged from 5 to 7, and the molecular weights (Mws) were mostly smaller than 30 kDa. The serum proteins showed no difference in pI or Mw for high- and low-flux HD. Gene ontology (GO) analysis showed that the detected proteins were related to immune system processes and complement activation.

**Conclusions:** Serum protein levels differentially changed after high- and low-flux HD. Long-term effects should be observed in future studies.

## Introduction

Hemodialysis (HD) is one of the most effective therapeutic methods for patients with uremia and is closely related to their outcomes. Three types of uremic toxins exist, including small molecular toxins, middle-weight toxins, and protein-binding toxins [1]. All three types of toxins can be removed to some extent during HD. Additionally, several proteins may be produced due to the contact of blood with dialysis membranes [2–4]. Currently, high-flux and low-flux HD are two routine modalities of clinical application. Thus, many studies have compared the similarities and differences between high-flux and low-flux HD with regard to different factors. Some large prospective clinical studies, such as the Haemodialysis (HEMO) study and the Membrane Permeability Outcome (MPO) Study, have failed to show a generally better outcome with high-flux HD than with low-flux HD treatment [5]. Many studies indicate that differences exist in the urea, phosphate, and β2-microglobulin (β2-MG) clearance [6] and in high-sensitivity C-reactive protein, blood glucose, blood insulin, interleukin-6 (IL-6), IL-8, and tumor necrosis factor α (TNF-α) levels between high-flux HD and low-flux HD treatment [7,8]. In some subgroups of HD patients, the use of high-flux membranes seems to improve the clinical outcome [9]. Some patients treated with high-flux HD had a reduced risk of cardiovascular complications and death [10,11]. Recently, proteomics methods have been successfully applied to the study of HD [6,12–17]. Therefore, we applied proteomics methods to identify the changes in serum proteins after the two forms of HD were performed and to identify the meaningful proteins to provide the basis for further research.

## Materials and methods

### Patients

Two patients were treated with both high-flux dialyzers (Polyflux 140H) and low-flux dialyzers (Polyflux 14L) during two continuous therapy sessions. These patients were maintained HD patients who underwent regular HD three times per week and were in a stable condition. The blood flow, dialysate flow rate, and treatment duration (4 h) were the same for both patients.

### Reagents

Dithiothreitol (DTT), iodoacetamide (IAA), trifluoroacetic acid (TFA), and formic acid (FA) were purchased from Sigma (St. Louis, MO, USA). Acetonitrile (ACN) and methanol were purchased from Merck (Darmstadt, Germany). Sequencing-grade modified trypsin was purchased from Promega (Madison, WI, USA). Deionized water was purified using a Milli-Q system (Millipore, Milford, MA, USA). A Microcon filtration device with a relative molecular weight (Mw) cutoff of 10,000 Da (10 K filter) was obtained from Sartorius AG (Gottingen, Germany). Other chemicals were of analytical grade. The C18 capillary column (75 μm i.d.×15 cm) was homemade.

### Sample preparation

Two milliliters of pre-dialysis blood were obtained before HD, and 2 mL of post-dialysis blood was obtained after each HD session was completed. The blood samples were stored in evacuated pro-coagulation tubes, followed by centrifugation at 3000 rpm for 10 min at room temperature. Then, the serum samples were transferred to centrifuge tubes, followed by centrifugation at 16 000 rpm for 20 min at 4 °C to separate the lipids. We diluted 10 μL of serum with 990 μL of 1× phosphate-buffered saline (PBS) and then transferred 150 μL of this liquid to a Microcon filtration device followed by centrifugation at 16 000 rpm for 10 min at 4 °C. Then, 200 μL of 1× PBS was added to each sample, and the samples were centrifuged at 16 000 rpm for 10 min at 4 °C. This step was repeated three times. The proteins were denatured with 90 μL of 1× PBS and 10 μL of 1 M DTT at 95 °C for 5 min and then washed with 200 μL of 1× PBS. Next, the proteins were subjected to alkylation with 20 mM IAA in the dark at room temperature for 40 min, followed by centrifugation at 16 000 rpm for 10 min. Finally, the samples were put in new collection tubes, and 6 μg of trypsin was added to the eight samples, which were incubated at 37 °C overnight, followed by washing with 100 μL of 1× PBS and centrifugation at 16 000 rpm for 10 min. The digested peptides were stored at –80 °C until use.

### Desalting

The serum protein samples were desalted by reverse-phase liquid chromatography using a Shimadzu LC-20AD HPLC system with a homemade C18 trap column (4.6 × 10 mm) at a flow rate 1 mL/min. Mobile phase A (98% H_2_O + 0.1% TFA) and mobile phase B (98% ACN + 0.1% TFA) were used for the gradient separation. Gradient elution was performed using 0% B (0–4 min), 80% B (4.1–14 min), and 0% B (14.1–20 min) [18]. Finally, the collected peptides were dried with a SpeedVac and stored at –80 °C until use.

### Dimethyl labeling

Tryptic peptides from pre-HD and post-HD samples were labeled with Cd_2_O and NaCNBH_3_ (heavy labeling, 32H) and with ^13^Cd_2_O and NaCNBD_3_ (light labeling, 32L), respectively [19,20]. Then, the mixture of labeled peptides was stored at 4 °C until subsequent use.

### Liquid chromatography-tandem mass spectrometry analysis

Liquid chromatography (Shimadzu LC-20AD) separation of the mixture of labeled peptides was performed on a C18 capillary column (75 μm i.d.×15 cm) with C18 silica particles (5 μm, 100 Å) under acidic conditions. The two eluent buffers used were H_2_O with 2% ACN and 0.1% FA (A) and 98% ACN with 2% H_2_O and 0.1% FA (B). The gradient of the mobile phase was set with the following parameters: 5–22% B for 120 min, 22–35% B for 30 min, and 35%–80% B for 5 min; maintenance with 80% B for 15 min and 2% B for 0.1 min; and maintenance with 2% B for 5 min at 0.5 mL/min. The resultant peptides were analyzed using a Q-Exactive Mass Spectrometer equipped with a Quaternary Surveyor pump and an ESI probe Ion Max Source with a microspray kit, controlled by Xcalibur software version 2.1.0 (Thermo Fisher, Waltham, MA, USA) in data-dependent acquisition mode. Full scan mass spectrometry (MS) spectra (from m/z 300–1800) were acquired in the orbitrap with a resolution of 70 000 at m/z 200. The 10 most intense ions with a charge ≥2 were subjected to high-collision fragmentation at a target value of 100 000 ions. The tandem mass spectra (MS/MS) were acquired in the orbitrap mass analyzer with a mass resolution of 17 500 at m/z 200 to identify adsorbed proteins. The dynamic exclusion time was set to 20 s, and the maximum allowed ion accumulation time was 50 ms for MS scans and 100 ms for MS/MS. The normalized collision energy was 28% for the second stage of MS (MS2) [18].

### Database searches

Raw data were processed with pFind2.0 (Institute of Computing Technology, Chinese Academy of Sciences, Beijing, China) against the Human UniProtKB database (Download: 12 December 2016; 71 242 proteins), and the parameters were set as follows [18]. Peptides were searched using fully tryptic cleavage constraints, and up to two cleavage sites were allowed for tryptic digestion. Protein N-terminal acetylation and methionine oxidation were set as variable modifications and cysteine carbamidomethylation was set as a static modification. Peptide identification was based on a search with an initial mass deviation of up to 10 ppm for the precursor ions and an allowed fragment mass deviation of 20 ppm. Heavy- and light-labeled samples were searched independently [19]. A false discovery rate (FDR) of 1% for both protein and peptide levels was required to filter the results [21]. The total intensities of the heavy and light paired fragment ions were used to calculate the ratio by pFind2.0 software [22,23].

### Statistical analysis

The statistical analysis was performed using SPSS version 22.0 (IBM Corporation, Chicago, IL, USA). The results are presented as medians ± interquartile range (IQR) for non-normally distributed data. Differences between groups for non-normally distributed variables were assessed with the Mann–Whitney U-test. The chi-square test was used to compare composition ratios. *p* values less than .05 were considered statistically significant.

### Bioinformatics analysis

The bioinformatics data were based on the gene ontology (GO) consortium and were assigned with PANTHER tools (http://www.pantherdb.org/).

## Results

After high-flux and low-flux HD, a total of 212 and 203 serum proteins, respectively, were identified with liquid chromatography-tandem mass spectrometry **(**LC-MS/MS) analysis. According to the ratio of chemical labeling (light labeling/high labeling), an increase or decrease was defined as an average ratio of >1 or <1, respectively. Insignificance was defined as a ratio >1 in one patient and <1 in the other patient. The isoelectric point (pI) values and Mw of the observed proteins were determined using the ‘Compute MW/pI’ tool from ExPASy (http://web.expasy.org/compute_pi/) and UniProt (http://www.uniprot.org/), respectively.

1. The amounts of serum proteins.

**Figure 1.**
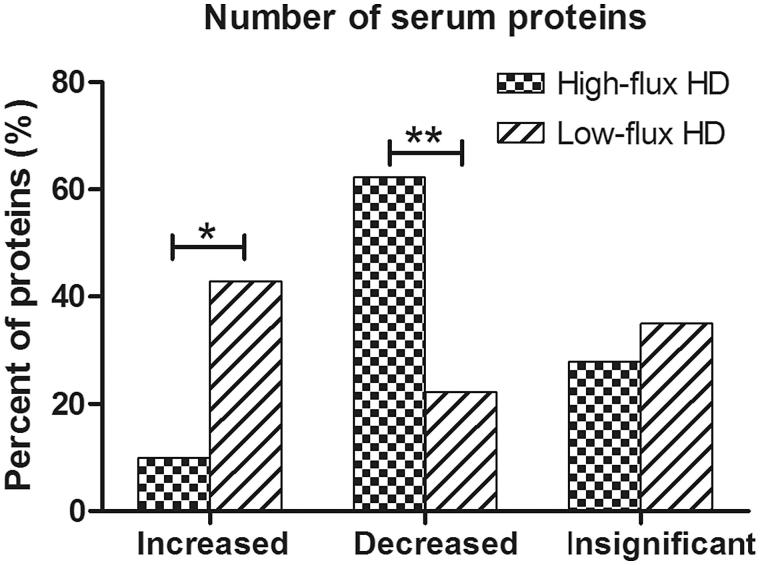
*: *p* < 0.05. The percentage of increased proteins was significantly different between high-flux HD and low-flux HD (9.9% vs. 42.9%, *χ*^2^ = 58.488, *p* = .000). **: *p* < .05. The percentage of decreased proteins was significantly different between high-flux HD and low-flux HD (62.3% vs. 22.2%, *χ*^2^ = 68.163, *p* = .000). The percentage of insignificant proteins was similar between high-flux HD and low-flux HD (27.8% vs. 35.0%, *χ*^2^ = 2.461, *p* = .117).

As shown in Figure 1, after high-flux HD, 21 proteins increased, 132 proteins decreased, and 59 proteins were insignificant. After low-flux HD, 87 proteins increased, 45 proteins decreased, and 71 proteins were insignificant. Nine proteins overlapped among the increased groups, and 20 proteins overlapped among the decreased groups (Table 1).

**Table 1.** Consensus serum proteins identified after high-flux and low-flux HD.

Entry	Name	Mw	pI
Increased			
P02042	Hemoglobin subunit delta	16 055	7.97
A0A0C4DH68	Protein IGKV2-24 (fragment)	13 079	8.01
P01782	Immunoglobulin heavy variable 3–9	12 945	6.85
D6RFL4	Monocyte differentiation antigen CD14 (fragment)	23 313	5
P01768	Immunoglobulin heavy variable 3–30	12 947	9.23
A0A0C4DH32	Protein IGHV3-20 (fragment)	12 673	8.08
Q5VY30	Retinol-binding protein 4, plasma, isoform CRA_b	22 974	5.77
P18428	Lipopolysaccharide-binding protein	53 384	6.25
A0A0B4J1X8	Immunoglobulin heavy variable 3–43	13 077	5.18
Decreased			
P31327	Carbamoyl-phosphate synthase [ammonia], mitochondrial	164 939	5.92
C9JXI5	Transmembrane protein 198 (fragment)	24 329	NS
K7ER74	Protein APOC4-APOC2	20 049	5.25
A0A0J9YXX1	Uncharacterized protein (fragment)	12 773	8.62
K7ERI9	Apolipoprotein C-I (fragment)	8647	6.13
C9JEE0	Immunoglobulin lambda-like polypeptide 1 (fragment)	19 227	NS
A0A0A0MSV6	Complement C1q subcomponent subunit B (fragment)	24 031	9.28
P43251	Biotinidase	61 133	5.38
P27169	Serum paraoxonase/arylesterase 1	39 731	5.08
P01008	Antithrombin-III	52 602	5.95
C9JF17	Apolipoprotein D (fragment)	24 158	5.2
P55056	Apolipoprotein C-IV	14 553	9.3
B4E1Z4	Uncharacterized protein	140 943	6.95
D6RF35	Vitamin D-binding protein	53 021	5.2
A0A075B6N9	Ig mu chain C region (fragment)	49 440	NS
P01780	Immunoglobulin heavy variable 3–7	12 943	8.65
V9GYM3	Apolipoprotein A-II	14 914	8.43
O95445	Apolipoprotein M	21 253	5.66
P02749	Beta-2-glycoprotein 1	38 298	8.37
H0YAC1	Plasma kallikrein (fragment)	76 885	NS

NS: no data were found with ExPasy.

2. The changes in protein levels after high-flux HD and low-flux HD.

**Figure 2.**
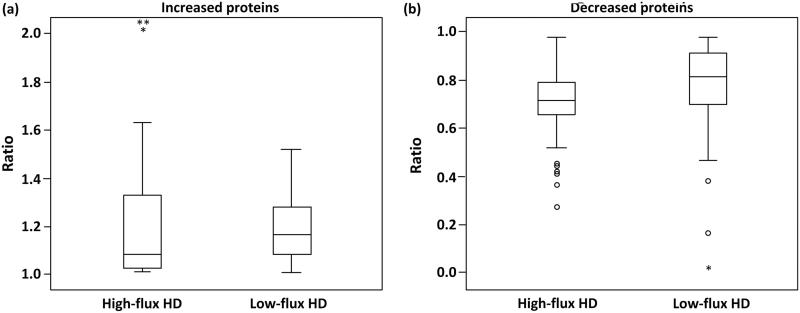
The ratios of proteins that increased and proteins that decreased were compared between high-flux HD and low-flux HD using the Mann–Whitney U-test. (a) Among the increased proteins, no significant difference was found (*p* > .05) between high-flux HD and low-flux HD (1.10 ± 0.32 vs. 1.17 ± 0.20, *p* = .23). (b) Among the decreased proteins, a significant difference was found (*p* < .05) between high-flux HD and low-flux HD (0.73 ± 0.13 vs. 0.84 ± 0.18, *p* = .00).

The ratios (after/before HD) of proteins are shown in Figure 2. Among the increased proteins, the average ratio was not significantly different between high-flux HD and low-flux HD. Among the decreased proteins, the average ratio after high-flux HD was significantly lower than the average ratio after low-flux HD (detailed data are shown in the supplementary table).

3. pI values and Mw of the serum proteins.

**Figure 3.**
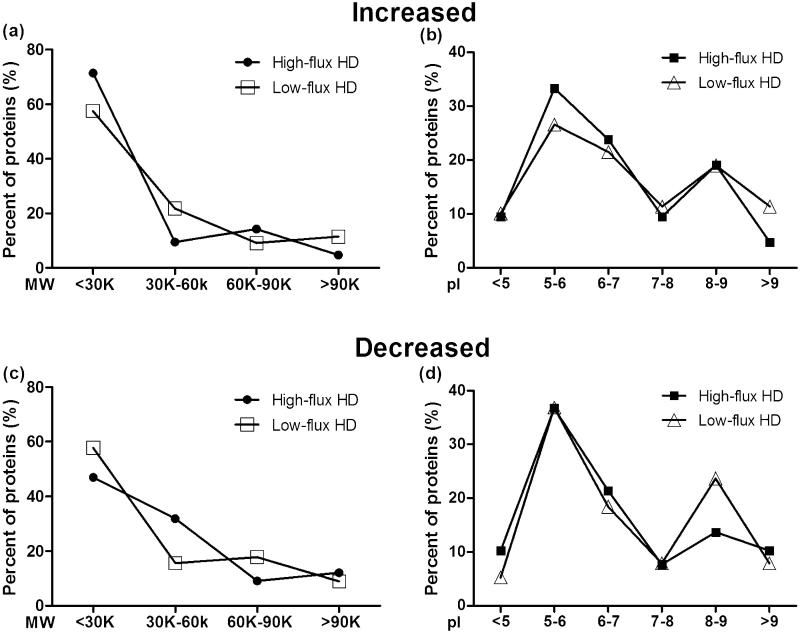
The percentages of proteins with various pI and Mw values were similar between the high-flux HD and low-flux HD groups for both increased and decreased proteins (chi-square test).

Among the increased and decreased proteins, the pI values mainly ranged from 5 to 7, and the Mws were mostly smaller than 30 kDa (Table 2). The serum proteins showed no difference in pI or Mw between high- and low-flux HD (Figure 3).

**Table 2.** pI values and Mw of detected proteins.

	Increased [*N* (%)]		Decreased [*N* (%)]	
	High-flux HD	Low-flux HD	High-flux HD	Low-flux HD
pI				
<5	2 (9.52%)	8 (10.13%)	12 (10.26%)	2 (5.26%)
5–6	7 (33.33%)	21 (26.85%)	43 (36.75%)	14 (36.84%)
6–7	5 (23.81%)	17 (21.52%)	25 (21.37%)	7 (18.42%)
7–8	2 (9.52%)	9 (11.39%)	9 (7.69%)	3 (7.89%)
8–9	4 (19.05%)	15 (18.99%)	16 (13.68%)	9 (23.68%)
>9	1 (4.76%)	9 (11.39%)	12 (10.26%)	3 (7.89%)
Mw				
<30 K	15 (71.43%)	50 (57.47%)	62 (46.97%)	26 (57.78%)
30–60 k	2 (9.52%)	19 (21.84%)	42 (31.82%)	7 (15.56%)
60–90 K	3 (14.29%)	8 (9.20%)	12 (9.09%)	8 (17.78%)
>90 K	1 (4.76%)	10 (11.49%)	16 (12.12%)	4 (8.89%)

4. GO analysis of the biological processes of detected proteins.

**Figure 4.**
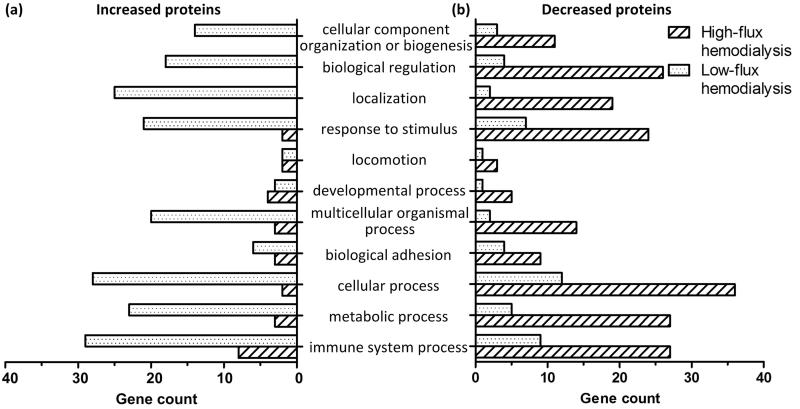
GO analysis of biological processes and gene counts of serum proteins.

PANTHER was used to analyze the biological processes of the detected proteins. The differences in gene count between high-flux HD and low-flux HD are shown in Figure 4. Among the increased proteins, the gene counts and GO terms of the biological processes in the low-flux HD group were much higher than those of the high-flux HD group. Among the decreased proteins, the GO terms of the high-flux HD group were similar to those of the low-flux HD group. However, the gene counts were much higher in the high-flux group than in the low-flux HD group.

5. GO enrichment analysis of the biological process of the detected proteins.

**Figure 5.**
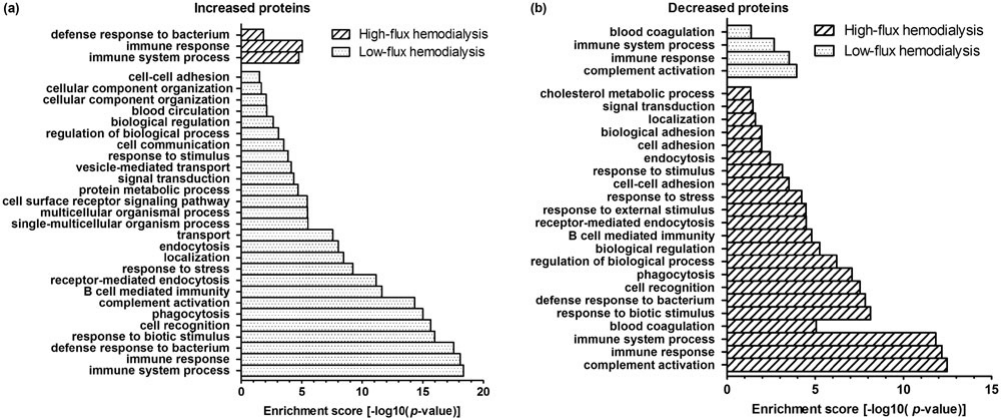
GO enrichment analysis of biological processes of the serum proteins.

GO enrichment analysis of the biological process of the detected proteins was also performed with PANTHER. A value of *p* < .05 was defined as significant. As shown in Figure 5, among the increased proteins, 27 GO terms were detected, and only 3 of the 27 GO terms were related to high-flux HD. Among the decreased proteins, 22 GO terms were detected, and only 4 of the 22 GO terms were related to low-flux HD.

## Discussion

In our study, no apparent difference was observed in the types of proteins identified after high-flux HD and low-flux HD. We found that more proteins decreased after high-flux HD, and more increased after low-flux HD. In our statistical analysis of the levels of increased and decreased proteins, although some proteins increased after both types of HD, the degree of change was not significantly different. This result indicates that these increased proteins only slightly influence the body although most were closely related to important biological functions, such as the immune system and activation of the complement system. Additionally, the degree by which proteins decreased after high-flux HD was significantly greater than that after low-flux HD, which indicates that high-flux HD removes proteins more effectively than low-flux HD. According to the pI and Mw analysis, a majority of increased and decreased proteins had a pI of 5–7 and an Mw <30 kDa. Thus, the two modalities of HD mainly impact proteins with a pI 5–7 and Mw <30 kDa, as no significant difference was found between high-flux HD and low-flux HD. Although the types of detected proteins were similar, the degree of reduction of proteins was different between the two types of HD. The proteins detected in our study were different from those detected by Hallbauer [24], and we could not confirm the long-term effectiveness of high- or low-flux HD treatment. In our bioinformatics analysis (Figures 4 and 5), the detected proteins (both increased and decreased) were related to important biological processes. The top three processes were immune system processes, immune response, and defense response to bacteria. This finding indicates that low-flux HD could induce the activation of some biological processes and high-flux HD could reduce activation, which reflects the biocompatibility of the dialyzers. This finding also suggests the bioincompatibility of low-flux dialyzers, which requires further investigation. Some studies have reported that the levels of several proteins differed after different types of HD. Laveborn [25] found that after low-flux HD, the biomarkers N-terminal-proBNP (NT-proBNP) and high-sensitivity cardiac troponin T (TnT) increased, whereas after high-flux HD, their levels decreased. Therefore, the elimination of biomarkers during HD should be a focus in clinical practice. When assessing the status of secondary parathyroid function in patients with uremia, the data gained from blood samples obtained after HD sessions might not be accurate, leading to inappropriate treatment. If the biomarkers TNT and NT-proBNP are influenced by different modalities of HD, as Laveborn described, the judgment of doctors will be affected to some extent, leading to inaccurate results.

As listed in Table 1, several proteins showed similar changes after high-flux HD and low-flux HD. Some proteins had important physiological functions, which could influence the human body. Dyslipidemia is characterized by increased plasma levels of atherogenic low-density lipoprotein cholesterol and reductions in anti-atherogenic high-density lipoprotein (HDL) cholesterol [26], which are the key pathogenic factors in atherosclerosis that lead to poor outcomes for maintained HD patients. However, the cause of dialysis-related disorders of lipid metabolism is not completely understood [27]. apoA-II is the second most abundant protein of the HDL particles and plays an important part in lipid metabolism. Low levels of HDL cholesterol and its main apoprotein constituents, apoA-I and apoA-II, are found in patients who are undergoing HD treatment [28]. Some studies have reported the significance of apoA-II in patients with chronic kidney disease or uremia. The mechanism underlying abnormalities in apoA-I and apoA-II involves an increased catabolic rate and a decreased production rate [29–31]. Other studies have shown that apolipoprotein C-I, apolipoprotein C-IV, apolipoprotein M, and apolipoprotein D are closely related to lipid metabolism [27,32,33]. Thus, the reduction in such proteins due to HD might be one cause of dyslipidemia in maintained HD patients. Vitamin D-binding protein (DBP) is mainly produced by the liver and has important physiological functions, including vitamin D transport and storage, actin scavenging, fatty acid transport and enhancement of the chemotactic activity of C5a in neutrophils during inflammation, and macrophage activation [34,35]. In addition, it plays an important role in the regulation of mineral and bone metabolism in patients with chronic kidney disease. Consequently, it has become a focus of renewed investigations concomitant with the surge of interest in vitamin D in recent years. Some studies have reported that a reduction in DBP is common in patients with nephrotic-range proteinuria, nephrotic syndrome, chronic kidney disease, or uremia, regardless of whether they are undergoing blood purification [36–39]. The main limitation of our study is the small number of samples. A larger number of samples will be investigated in our future studies.

## Conclusions

Serum protein levels changed after high-flux HD. More serum proteins tended to increase after low-flux HD while more tended to decrease after high-flux HD. The levels of increased proteins were not significantly different between high- and low-flux HD while the levels of decreased proteins were different. Compared to low-flux HD, high-flux HD leads to an apparent reduction in some proteins. Moreover, these differences were not correlated with the pI or Mw of the proteins. These proteins participate in important biological processes, such as immune system processes, the immune response, and complement activation. Therefore, low-flux HD might induce such biological reactions, whereas high-flux HD reduces these reactions. Several proteins with important physical functions, such as apoA-II and DBP, decreased after HD and this should be noted in clinical practice. The long-term effects of these changes should be observed in future studies.

## Supplementary Material

Supplementary file
